# Association between Serum Cholesterol Level and Osteoporotic Fractures

**DOI:** 10.3389/fendo.2018.00030

**Published:** 2018-02-12

**Authors:** Yanmao Wang, Jiezhi Dai, Wanrun Zhong, Chengfang Hu, Shengdi Lu, Yimin Chai

**Affiliations:** ^1^Department of Orthopedic Surgery, Shanghai Jiao Tong University Affiliated Sixth People’s Hospital, Shanghai, China

**Keywords:** serum cholesterol, high-density lipoprotein, fracture, cross-sectional studies, risk factor

## Abstract

**Objective:**

Previous epidemiological studies have found an association between serum cholesterol level and bone mineral density. However, epidemiological studies evaluating the association between serum cholesterol level and the incidence of osteoporotic fracture are scant. Therefore, the objective of this study was to investigate whether serum cholesterol levels in Chinese participants aged 55 years or older was associated with an increased risk of osteoporotic fracture.

**Materials and methods:**

We performed a cross-sectional study, including 1,791 participants (62.1% postmenopausal women and 213 fractures). Standardized self-administered questionnaires, physical examination, laboratory tests, and dual-energy X-ray absorptiometry examination were performed. Multivariate-adjusted logistic regression models were used to evaluate associations between serum cholesterol [total cholesterol (TC), triglycerides (TG), high-density lipoprotein (HDL-C), and low-density lipoprotein (LDL-C)] levels and the osteoporotic fracture risk.

**Results:**

After adjusting for potential confounding factors, there were no associations between per SD increase in TC and LDL level and an increased risk of osteoporotic fracture in total participants, and in men and women as individual groups. There was a significant association between per SD increase in HDL-C level and an increased risk of osteoporotic fracture in total participants [odds ratios (OR) 1.20, 95% confidence interval (CI) 1.03, 1.40, *P* = 0.023] and in women (OR 1.37, 95% CI 1.12, 1.68, *P* = 0.003), whereas no association was observed in men (OR 1.01, 95% CI 0.73, 1.40, *P* = 0.951). Additionally, we found a significant association between per SD increase in TG level and an increased risk of osteoporotic fracture in total participants (OR 1.20, 95% CI 1.04, 1.38, *P* = 0.015). In women, a nonlinear relationship was observed between per SD increase in TG level and an increased risk of osteoporotic fracture. The risk of osteoporotic fracture in women increased with TG level >1.64 mmol/L (OR 1.93, 95% CI 1.24, 3.00, *P* = 0.004).

**Conclusion:**

Among Chinese older adults, serum HDL-C level is significantly associated with a risk of osteoporotic fractures in women, and serum TG level is significantly associated with a risk of osteoporotic fractures in total participants and in women with TG >1.64 mmol/L.

## Introduction

Osteoporosis, a multiple system-related disease with bone loss and bone microstructure damage and change, has a high incidence throughout the world (1, 2). In 2010, there were approximately 6% of men and 21% of women (total 27.6 million patients) aged 50–84 years who were diagnosed with osteoporosis in Europe. More than 8.9 million osteoporosis-related fractures occurred annually worldwide and over one-third of these fractures occurred in Europe (1–3). Moreover, A previous study established certain risk factors for osteoporosis and osteoporotic fractures, such as lifestyle and diet, history of diseases and medication, and genetic factors (4–6). In recent years, the link between lipid levels, metabolism, and osteoporosis has been better understood (7–10). Bone mineral density (BMD) has been reported to be associated with metabolic syndrome and cardiovascular events (11–16), while the lipid profile may have an important role in the aforementioned link (11–16).

Many observational studies have investigated the association between serum cholesterol [total cholesterol (TC), triglycerides (TG), high-density lipoprotein (HDL-C), and low-density lipoprotein (LDL-C)] level and BMD. However, the results are controversial (8, 9, 17–26). In a Northern Sydney Twin Study, Makovey et al. found an inverse relationship between serum TC and LDL levels and lumbar spine and whole body BMD in postmenopausal women as well as between HDL levels and BMD at all sites in premenopausal women (9). Another cohort including 1,289 African ancestry men reported that lower TG or LDL and higher HDL status were associated with lower trabecular BMD (27). Moreover, a cross-sectional and a longitudinal study that included 958 Korean postmenopausal women found no association between serum lipid profiles and BMD in postmenopausal women (20).

However, only few observational studies have investigated the association between serum cholesterol level and fractures (28, 29). Trimpou et al. preformed a prospective study form the Gothenburg WHO MONICA project that included 1,396 participants. In doing so, they found serum TC is an independent osteoporotic fracture risk factor (28). Another epidemiological study of 211 healthy Japanese postmenopausal women (age range, 46–80 years) performed by Yamauchi et al. investigated the associations between serum LDL-C level and BMD, and suggested that a high-serum LDL-C level may be a risk factor for prevalent non-vertebral fragility fractures, after adjustment for age, body mass index (BMI), years since menopause, physical activity, previous cardiovascular events, bone markers, BMD, serum Ca, P, Cr, 25(OH)D, grip strength, tandem gait test, and use of drugs for hyperlipidemia (29).

The objective of this study was to investigate whether serum cholesterol (TC, TG, HDL-C, and LDL-C) level in men and women 55 years of age or older is associated with osteoporotic fracture risk in the Chinese population. We further sought to explore whether there are other interactions that affect this association. Since sex may affect the real association between serum cholesterol level and osteoporotic fracture, we performed analyses separately in men and women.

## Subjects and Methods

### Study Population and Design

This was a cross-sectional study of 3,891 participants aged 55–85 years of age which who were recruited from the Xuhui and Yangpu districts of the Shanghai metropolitan area, China, between June 2014 and June 2016. There were 3,657 participants who had completed self-administered questionnaires, physical examinations, and laboratory tests. The exclusion criteria were as follows: (a) history of hepatitis, liver cirrhosis, any cancer or malignancy (*n* = 285); (b) history of kidney disease (*n* = 82); (c) regular treatment for osteoporosis (*n* = 192); (d) excessive alcohol consumption of greater than or equal to 140 g/week for men or 70 g/week for women (*n* = 311); (e) history of diabetes mellitus (*n* = 428); (f) history of rheumatoid arthritis (*n* = 334); (g) history of thyroid diseases (*n* = 172); or (e) absence of baseline data (*n* = 62). Thus, 1,791 participants (679 men and 1,112 women) were eventually included in this analysis (Figure S1 in Supplementary Material). This study was approved by the Ethics Committee of Shanghai Sixth People’s Hospital, and all participants provided written informed consent.

### Participant Characteristics

Physicians were trained to assist participants in completing the standardized self-administered questionnaires (including demographic characteristics, lifestyle, calcium or vitamin D supplementation, etc.). Chronic disease history (including cardiovascular disease, diabetes, hypertension, chronic kidney disease, dyslipidemia, fracture, and other conditions) and medication history (especially anti-osteoporosis treatment) were also recorded. Smoking and alcohol status was defined as either never, current, or previously used. The type, frequency, and dose of alcohol consumption were collected and the level of mean daily alcohol drinking was calculated. Physical activity at leisure time was classified as less than 30 min/day, 30–60 min/day, 1–2 h/day, 2–4 h/day, 4–6 h/day, or more than 6 h/day.

Physical examinations were evaluated according to standardized protocol and by the trained inspectors. Standing height and body weight were measured while participants were wearing lightweight clothing and no shoes. Blood pressure, including systolic blood pressure (SBP) and diastolic blood pressure (DBP), was measured with an automatic sphygmomanometer, and the participants were asked to rest at least 5 min before examination. BMI used as an index of body fat, was calculated as weight in kilograms divided by height in meters squared (kg/m^2^). Waist circumference (WC) was measured horizontally at the umbilicus level. Total hip BMD was measured by dual-energy X-ray absorptiometry using a Lunar Prodigy GE densitometer (Lunar Corp, Madison, WI, USA). We calculated the T-score by comparing the values recorded here with the average peak hip BMD of young, healthy Chinese in the same study area between 25 and 30 years of age.

Laboratory tests included measurements of TG, TC, HDL-c, LDL-c, glucose, and calcium levels. All values were measured *via* an automated analyzer using standard methods. Hypercholesterolemia was defined as TC level ≥5.0 mmol/L and hypertriglyceridemia was defined as TG level ≥1.7 mmol/L (30, 31). Metabolic syndrome was defined according to the reported harmonized criteria (32). People with three or more of the following criteria were considered to have metabolic syndrome: (1) waist circumference ≥90 cm in men or ≥80 cm in women; (2) TG ≥150 mg/dL; (3) HDL-C <40 mg/dL in men or <50 mg/dL in women; (4) elevated SBP ≥130 mmHg and/or DBP ≥85 mmHg or use of anti-hypertensive medications; or (5) blood glucose ≥100 mg/dL or receiving treatment for diabetes.

### Assessment of Fractures

According to interviewer-assisted standardized self-administered questionnaires, the history of fractures was collected, including fracture sites and the age at which the fractures occurred. In this study, osteoporotic fractures were defined as low trauma fractures (33, 34). All pathologic (due to cancer, bone tuberculosis, etc.) or traumatic fractures (defined as a fracture that resulted from a fall greater than a standing height, or accident including motor vehicle accident, sports accident, etc.) were excluded (33, 34).

### Statistical Analysis

Since sex may be an important factor that influences the association between serum cholesterol level and osteoporotic fracture, we performed analyses separately in men and women. Continuous variables are presented as the mean ± SD, while categorical variables are presented as the number and proportion. To compare differences between groups, we used chi-square tests for categorical variables, one-way ANOVA for normally continuous variables, and the Kruskal–Wallis test for skewed continuous variables. We calculated odds ratios (OR) and corresponding 95% confidence interval (CI) using logistic regression analyses for the associations between per SD increase in serum cholesterol level and osteoporotic fracture. Unadjusted and multivariate adjusted logistic regression analyses were performed. Model 1 was adjusted for age; Model 2 was further adjusted for BMI, and waistline based on Model 1; Model 3 was further adjusted for smoking status, alcohol status (never/ever or current), physical activity (<30 min a day/0.5–1 h a day/ >1 h a day) based on Model 2; Model 4 was further adjusted for hypertension, cardiovascular events, metabolic syndrome, and family history of hip fracture based on Model 3; Model 5 was further adjusted for blood glucose and blood Ca based on Model 4; Model 6 was further adjusted for calcium and vitamin D supplementation based on Model 5; and Model 7 was further adjusted for T-score for total hip based on Model 6.

To examine the nonlinear or threshold effect of the association between serum cholesterol level and osteoporotic fracture (logOR), we further applied a two-piecewise linear regression model using a smoothing function after adjusting for Model 7. The threshold level was determined using trial and error, including selection of turning points along a predefined interval and then choosing the turning point that gave the maximum-likelihood model. We also conducted a log-likelihood ratio test comparing the one-line linear regression model with a two-piecewise linear model.

A two-sided *P*-value <0.05 was considered significant. Statistical analyses were performed using PASW Statistics 18.0 software (SPSS Inc., Chicago, IL, USA), Empower (R)^1^ (X&Y Solutions Inc., Boston, MA, USA) and R packages.^2^

## Results

### Participant Characteristics

Of the 1,791 participants (679 men and 1,112 women) included in this analysis, 213 had fractures (58 lumbar fractures, 29 radius fractures, 23 hip fractures, and 103 other fractures). The clinical and laboratory characteristics stratified by fracture are shown in Table 1. In men, there were no differences in age, BMI, waistline, blood pressure, T-score for hip, or serum TC, TG, HDL-C, or LDL-C levels (all *P* > 0.05). In women, participants with fracture had a higher BMI (24.17 ± 4.33 vs. 23.55 ± 3.47 kg/m^2^, *P* = 0.049), lower T-score for hip (−2.19 ± 1.11 vs. −1.97 ± 1.03, *P* = 0.011), and higher HDL-C level (1.61 ± 0.38 vs. 1.54 ± 0.35 mmol/L, *P* = 0.034) than did participants without fractures. Moreover, in women, there were no differences in age, waistline, blood pressure, and serum TC, TG or LDL-C (all >0.05) levels. The prevalence of hypertension and cardiovascular events were similar in all groups.

**Table 1:** Characteristics of participants stratified by sex.

	Total participants (*n* = 1,791)			Men (*n* = 679)			Women (1,112)		
	Fracture (−)	Fracture (+)	*P*-value	Fracture (−)	Fracture (+)	*P*-value	Fracture (−)	Fracture (+)	*P*-value
*N*	1,578	213		624	55		954	158	
Age (years)	73.14 ± 6.13	73.37 ± 5.81	0.617	74.30 ± 6.03	73.98 ± 5.99	0.707	72.39 ± 6.08	73.15 ± 5.75	0.140
BMI (kg/m^2^)	23.61 ± 3.32	24.15 ± 4.09	0.137	23.70 ± 3.08	24.11 ± 3.33	0.347	23.55 ± 3.47	24.17 ± 4.33	0.049
Waistline (cm)	83.03 ± 8.93	82.78 ± 11.30	0.715	85.81 ± 8.94	87.31 ± 12.57	0.251	81.20 ± 8.44	81.16 ± 10.39	0.956
Diastolic pressure	129.48 ± 15.87	128.70 ± 14.66	0.501	131.13 ± 15.07	129.20 ± 16.02	0.366	128.40 ± 16.30	128.53 ± 14.20	0.931
Systolic pressure	79.37 ± 9.69	79.65 ± 7.57	0.693	80.93 ± 10.05	81.60 ± 7.81	0.633	78.34 ± 9.30	78.95 ± 7.37	0.442
T-score of hip	−1.79 ± 1.07	−2.05 ± 1.12	0.001	−1.51 ± 1.06	−1.60 ± 1.06	0.540	−1.97 ± 1.03	−2.19 ± 1.11	0.011
TC (mmol/L)	5.19 ± 0.96	5.36 ± 1.05	0.023	4.97 ± 0.91	5.00 ± 1.15	0.853	5.34 ± 0.96	5.49 ± 0.98	0.094
TG (mmol/L)	1.70 ± 0.93	1.84 ± 1.23	0.263	1.64 ± 0.91	1.76 ± 1.04	0.395	1.74 ± 0.94	1.87 ± 1.29	0.116
HDL-C (mmol/L)	1.48 ± 0.36	1.56 ± 0.37	0.002	1.38 ± 0.36	1.43 ± 0.31	0.325	1.54 ± 0.35	1.61 ± 0.38	0.034
LDL-C (mmol/L)	2.81 ± 0.72	2.89 ± 0.76	0.111	2.68 ± 0.69	2.69 ± 0.94	0.979	2.89 ± 0.73	2.97 ± 0.68	0.232
Glucose (mmol/L)	5.17 ± 1.05	5.10 ± 0.95	0.371	5.25 ± 1.06	4.85 ± 0.55	0.008	5.12 ± 1.03	5.19 ± 1.04	0.434
Ca (mmol/L)	2.25 ± 0.29	2.25 ± 0.34	0.958	2.24 ± 0.32	2.18 ± 0.26	0.209	2.25 ± 0.28	2.27 ± 0.36	0.443
Hypertension			0.251			0.458			0.170
No	973 (61.66%)	140 (65.73%)		350 (56.09%)	28 (50.91%)		623 (65.30%)	112 (70.89%)	
Yes	605 (38.34%)	73 (34.27%)		274 (43.91%)	27 (49.09%)		331 (34.70%)	46 (29.11%)	
Cardiovascular events			0.036			0.145			0.089
No	1,248 (79.09%)	155 (72.77%)		485 (77.72%)	38 (69.09%)		763 (79.98%)	117 (74.05%)	
Yes	330 (20.91%)	58 (27.23%)		139 (22.28%)	17 (30.91%)		191 (20.02%)	41 (25.95%)	
Family history of fracture			0.043			0.147			0.197
No	1,259 (87.25%)	170 (82.13%)		497 (89.55%)	44 (83.02%)		762 (85.81%)	126 (81.82%)	
Yes	184 (12.75%)	37 (17.87%)		58 (10.45%)	9 (16.98%)		126 (14.19%)	28 (18.18%)	
Smoke status			0.020			0.741			0.729
Never	1,247 (79.12%)	183 (85.92%)		314 (50.40%)	29 (52.73%)		933 (97.90%)	154 (97.47%)	
Ever or current	329 (20.88%)	30 (14.08%)		309 (49.60%)	26 (47.27%)		20 (2.10%)	4 (2.53%)	
Alcohol status			0.157			0.377			0.875
Never	1,296 (82.86%)	183 (86.73%)		377 (60.90%)	29 (54.72%)		919 (97.25%)	154 (97.47%)	
Ever or current	268 (17.14%)	28 (13.27%)		242 (39.10%)	24 (45.28%)		26 (2.75%)	4 (2.53%)	
Physical activity			0.946			0.366			0.595
<30 min/day	405 (25.88%)	53 (24.88%)		154 (24.88%)	16 (29.09%)		251 (26.53%)	37 (23.42%)	
0.5–1 h/day	596 (38.08%)	83 (38.97%)		237 (38.29%)	24 (43.64%)		359 (37.95%)	59 (37.34%)	
>1 h/day	564 (36.04%)	77 (36.15%)		228 (36.83%)	15 (27.27%)		336 (35.52%)	62 (39.24%)	
TC			0.176			0.361			0.414
≤5.0 mmol/L	1,349 (88.00%)	187 (91.22%)		319 (51.95%)	31 (58.49%)		328 (35.65%)	49 (32.24%)	
>5.0 mmol/L	184 (12.00%)	18 (8.78%)		295 (48.05%)	22 (41.51%)		592 (64.35%)	103 (67.76%)	
TG			0.318			0.089			0.962
≤1.7 mmol/L	968 (63.10%)	122 (59.51%)		407 (66.29%)	29 (54.72%)		561 (60.98%)	93 (61.18%)	
>1.7 mmol/L	566 (36.90%)	83 (40.49%)		207 (33.71%)	24 (45.28%)		359 (39.02%)	59 (38.82%)	

*Data are means ± SD or numbers (percent). *P*-values were calculated from chi-squared test for categorical variables, the one-way ANOVA for normally distributed continuous variables, and the Kruskale–Wallis test for skewed continuous variables*.

### The Association between Serum Cholesterol Level and Osteoporotic Fracture

Table 2 shows the association between serum cholesterol level and osteoporotic fracture. For serum TC, in the unadjusted model, there was a significant association between per SD increase in serum TC levels, and osteoporotic fractures in total participants (OR 1.18, 95% CI 1.02, 1.37; *P* = 0.023). After adjusting for sex, age, smoking status (never/ever or current), alcohol status (never/ever or current), BMI, waistline, physical activity (<30 min a day/0.5–1 h a day/>1 h a day), hypertension, cardiovascular events, metabolic syndrome, family history of hip fracture, blood glucose, blood Ca calcium supplement, vitamin D supplement, and T-score for total hip (Model 7), this association changed (OR 1.14, 95% CI 0.97, 1.36; *P* = 0.121). Moreover, we did not observe a nonlinear association between serum TC and osteoporotic fracture (Table S1 and Figure S2 in Supplementary Material, all *P* for nonlinear >0.05).

**Table 2:** Association between TC, TG, HDL-C, LDL-C, and osteoporotic fracture.

	Total participants		Men		Women	
	OR (95%CI)	*P*-value	OR (95%CI)	*P*-value	OR (95%CI)	*P*-value
**TC, per SD increase**						
Unadjusted	1.18 (1.02, 1.37)	0.0228	1.03 (0.77, 1.38)	0.8528	1.16 (0.98, 1.38)	0.0938
Model 1	1.13 (0.97, 1.30)	0.1194	1.02 (0.77, 1.36)	0.8856	1.16 (0.98, 1.38)	0.0907
Model 2	1.14 (0.98, 1.33)	0.0840	1.02 (0.76, 1.37)	0.8909	1.19 (1.00, 1.42)	0.0516
Model 3	1.14 (0.98, 1.33)	0.0835	1.01 (0.75, 1.35)	0.9610	1.19 (1.00, 1.42)	0.0552
Model 4	1.13 (0.96, 1.32)	0.1385	1.08 (0.81, 1.45)	0.6006	1.15 (0.95, 1.39)	0.1465
Model 5	1.11 (0.95, 1.31)	0.1927	1.18 (0.86, 1.61)	0.3072	1.13 (0.93, 1.37)	0.2283
Model 6	1.15 (0.97, 1.36)	0.1190	1.11 (0.80, 1.54)	0.5142	1.19 (0.96, 1.47)	0.1071
Model 7	1.14 (0.97, 1.36)	0.1207	1.03 (0.73, 1.44)	0.8814	1.20 (0.97, 1.49)	0.0864

**TG, per SD increase**						
Unadjusted	1.14 (1.00, 1.30)	0.0470	1.12 (0.86, 1.45)	0.3953	1.13 (0.97, 1.31)	0.1173
Model 1	1.13 (0.99, 1.29)	0.0651	1.11 (0.85, 1.44)	0.4472	1.14 (0.98, 1.32)	0.1016
Model 2	1.14 (1.00, 1.31)	0.0576	1.09 (0.83, 1.43)	0.5347	1.16 (0.99, 1.36)	0.0656
Model 3	1.14 (0.99, 1.30)	0.0639	1.08 (0.82, 1.42)	0.6002	1.16 (0.99, 1.35)	0.0734
Model 4	1.13 (0.97, 1.30)	0.1088	1.13 (0.85, 1.50)	0.3971	1.13 (0.95, 1.33)	0.1755
Model 5	1.11 (0.96, 1.29)	0.1664	1.13 (0.86, 1.49)	0.3860	1.09 (0.92, 1.31)	0.3256
Model 6	1.16 (1.00, 1.36)	0.0521	1.16 (0.88, 1.52)	0.2942	1.14 (0.95, 1.38)	0.1675
Model 7	1.20 (1.04, 1.38)	0.0153	1.16 (0.88, 1.54)	0.2932	1.14 (0.95, 1.38)	0.1683

**HDL-C, per SD increase**						
Unadjusted	1.23 (1.08, 1.40)	0.0025	1.12 (0.89, 1.42)	0.3283	1.20 (1.01, 1.43)	0.0340
Model 1	1.17 (1.02, 1.34)	0.0212	1.12 (0.89, 1.41)	0.3313	1.20 (1.01, 1.42)	0.0365
Model 2	1.21 (1.05, 1.39)	0.0084	1.14 (0.91, 1.43)	0.2545	1.24 (1.04, 1.48)	0.0188
Model 3	1.21 (1.05, 1.39)	0.0089	1.13 (0.90, 1.42)	0.2938	1.25 (1.04, 1.49)	0.0164
Model 4	1.20 (1.04, 1.39)	0.0145	1.13 (0.89, 1.44)	0.3176	1.25 (1.03, 1.50)	0.0207
Model 5	1.21 (1.04, 1.40)	0.0124	1.13 (0.88, 1.46)	0.3395	1.31 (1.08, 1.59)	0.0059
Model 6	1.21 (1.04, 1.41)	0.0162	1.04 (0.77, 1.41)	0.7959	1.37 (1.12, 1.68)	0.0026
Model 7	1.20 (1.03, 1.40)	0.0229	1.01 (0.73, 1.40)	0.9505	1.37 (1.12, 1.68)	0.0027

**LDL-C, per SD increase**						
Unadjusted	1.12 (0.97, 1.29)	0.1115	1.00 (0.75, 1.34)	0.9790	1.11 (0.94, 1.31)	0.2318
Model 1	1.08 (0.93, 1.25)	0.2963	1.00 (0.75, 1.33)	0.9944	1.11 (0.93, 1.31)	0.2392
Model 2	1.07 (0.93, 1.24)	0.3503	0.98 (0.74, 1.32)	0.9155	1.10 (0.93, 1.31)	0.2699
Model 3	1.07 (0.93, 1.24)	0.3529	0.97 (0.72, 1.31)	0.8536	1.10 (0.92, 1.30)	0.2933
Model 4	1.08 (0.93, 1.26)	0.3077	1.04 (0.78, 1.39)	0.7968	1.09 (0.91, 1.31)	0.3545
Model 5	1.08 (0.92, 1.26)	0.3340	1.06 (0.79, 1.42)	0.7101	1.08 (0.90, 1.31)	0.4190
Model 6	1.08 (0.92, 1.27)	0.3568	0.97 (0.72, 1.32)	0.8587	1.10 (0.90, 1.34)	0.3330
Model 7	1.06 (0.90, 1.25)	0.4668	0.92 (0.67, 1.25)	0.5860	1.10 (0.90, 1.34)	0.3458

*Data are OR (95% CI). Model 1 was adjusted for age (age and sex for total participants); Model 2 was further adjusted for BMI, and waistline based on Model 1; Model 3 was further adjusted for smoking status, alcohol status (never/ever or current), physical activity (<30 min a day/0.5–1 h a day/>1 h a day) based on Model 2; Model 4 was further adjusted for hypertension, cardiovascular events, metabolic syndrome, and family history of hip fracture based on Model 3; Model 5 was further adjusted for blood glucose and blood Ca based on Model 4; Model 6 was further adjusted for calcium and vitamin D supplementation based on Model 5; Model 7 was further adjusted for T-score for total hip based on Model 6*.

For serum TG, in both unadjusted Models and 7, there were significant association between per SD increase in serum TG levels, and osteoporotic fractures in total participants (unadjusted model: OR 1.14, 95% CI 1.00, 1.30; *P* = 0.047, Model 7: OR 1.20, 95% CI 1.04, 1.38; *P* = 0.015). However, in both men and women, no significant association was found between per SD increase in serum TG levels and osteoporotic fractures. In women, a nonlinear relationship was observed between per SD increase in TG level and an increased risk of osteoporotic fracture. The osteoporotic fracture in women increased with TG >1.64 mmol/L (Table S1 and Figure S2 in Supplementary Material, OR 1.93, 95% CI 1.24, 3.00, *P* for nonlinear = 0.013).

For serum HDL-C, there was a significant association between per SD increase in HDL-C level and an increased risk of osteoporotic fracture in total participants (OR 1.20, 95% CI 1.03, 1.40, *P* = 0.023) and in women (OR 1.37, 95% CI 1.12, 1.68, *P* = 0.003), whereas no association was observed in men (OR 1.01, 95% CI 0.73, 1.40, *P* = 0.951), after adjusting for Model 7. Moreover, there was no nonlinear association between serum HDL-C and osteoporotic fracture (Table S1 and Figure S2 in Supplementary Material, all *P* for nonlinear >0.05).

For serum LDL-C, in both the unadjusted Models and 7, there were no associations between per SD increase in LDL level and an increased risk of osteoporotic fracture in all participants as well as in men and women as separate groups (all *P* > 0.05). Moreover, there was no nonlinear association between serum HDL-C and osteoporotic fracture. (Table S1 and Figure S2 in Supplementary Material, all *P* for nonlinear >0.05)

### The Association between Serum Cholesterol Level and Fracture Type

Table 3 shows the association between serum cholesterol level and osteoporotic fracture type. For serum TC, there was a significant association between per SD increase in serum TC levels and lumbar fracture in all participants and in women alone, whereas no association was observed in men. However, there was a significant association between per SD increase in serum TC levels and radius fracture or hip fracture. For serum TG, there was a significant association between per SD increase in serum TG levels and radius fracture or lumbar fracture in women, whereas no association was observed in men. Moreover, there were no significant associations between per SD increase in serum HDL-C levels or LDL-C and radius fracture, lumbar fracture, or hip fracture in both men and women.

**Table 3:** Association between TC, TG, HDL-C, LDL-C and osteoporotic fracture, lumbar fracture, radius fracture, and hip fracture.

	Total participants		Men		Women	
	OR (95%CI)	*P*-value	OR (95%CI)	*P*-value	OR (95%CI)	*P*-value
**Any fracture**						
TC, per SD increase	1.14 (0.97, 1.36)	0.1207	1.03 (0.73, 1.44)	0.8814	1.20 (0.97, 1.49)	0.0864
TG, per SD increase	1.20 (1.04, 1.38)	0.0153	1.16 (0.88, 1.54)	0.2932	1.14 (0.95, 1.38)	0.1683
HDL-C, per SD increase	1.20 (1.03, 1.40)	0.0229	1.01 (0.73, 1.40)	0.9505	1.37 (1.12, 1.68)	0.0027
LDL-C, per SD increase	1.06 (0.90, 1.25)	0.4668	0.92 (0.67, 1.25)	0.5860	1.10 (0.90, 1.34)	0.3458

**Lumbar fracture**						
TC, per SD increase	1.53 (1.10, 2.11)	0.0103	1.44 (0.52, 4.01)	0.4796	1.64 (1.14, 2.36)	0.0073
TG, per SD increase	1.26 (0.97, 1.65)	0.0832	0.18 (0.02, 1.41)	0.1031	1.44 (1.08, 1.91)	0.0130
HDL-C, per SD increase	1.23 (0.93, 1.61)	0.1412	2.70 (0.93, 7.87)	0.0682	1.25 (0.87, 1.81)	0.2285
LDL-C, per SD increase	1.25 (0.92, 1.69)	0.1534	1.54 (0.57, 4.19)	0.3967	1.23 (0.87, 1.73)	0.2397

**Radius fracture**						
TC, per SD increase	0.94 (0.62, 1.42)	0.7690	0.75 (0.33, 1.71)	0.4955	1.00 (0.58, 1.72)	0.9968
TG, per SD increase	1.42 (1.06, 1.90)	0.0180	0.67 (0.25, 1.79)	0.4195	2.04 (1.38, 3.00)	0.0003
HDL-C, per SD increase	0.95 (0.62, 1.47)	0.8337	0.96 (0.45, 2.07)	0.9164	1.09 (0.62, 1.90)	0.7650
LDL-C, per SD increase	0.99 (0.65, 1.50)	0.9484	0.79 (0.36, 1.69)	0.5359	0.95 (0.57, 1.59)	0.8548

**Hip fracture**						
TC, per SD increase	1.51 (0.89, 2.55)	0.1262	NA		1.19 (0.64, 2.19)	0.5796
TG, per SD increase	0.91 (0.51, 1.63)	0.7456	3.67 (0.76, 17.82)	0.1067	0.54 (0.23, 1.26)	0.1553
HDL-C, per SD increase	1.22 (0.78, 1.91)	0.3809	2.07 (0.41, 10.48)	0.3772	1.21 (0.68, 2.18)	0.5140
LDL-C, per SD increase	1.73 (1.09, 2.73)	0.0194	30.17 (0.19, 4916.16)	0.1898	1.67 (0.99, 2.82)	0.0536

*Data are OR (95% CI). Adjusted for sex (only for total participants), age, smoking status (never/ever or current), alcohol status (never/ever or current), BMI, waistline, physical activity (<30 min a day/0.5–1 h a day/>1 h a day), hypertension, cardiovascular events, metabolic syndrome, family history of hip fracture, blood glucose, blood Ca, calcium and vitamin D supplementation, and T-score for total hip*.

### Impact of Hypertension and Cardiovascular Events on the Association between Serum Cholesterol Level and Osteoporotic Fracture

We examined whether hypertension and cardiovascular events can impact the association between per SD increase in serum cholesterol level and osteoporotic fracture. As shown in Table 4, the significant interactive effect was not detected in both men and women (all *P*-interaction >0.05).

**Table 4:** Effect of cardiovascular events and hypertension on the association between TC, TG, HDL-C, LDL-C, and osteoporotic fracture.

	Total participants			Men			Women		
	OR (95%CI)	*P*-value	*P*-value*	OR (95%CI)	*P*-value	*P*-value*	OR (95%CI)	*P*-value	*P*-value*
**TC, per SD increase**									
Cardiovascular events									
No	1.27 (1.03, 1.57)	0.0252	0.0526	1.04 (0.66, 1.63)	0.8693	0.9583	1.38 (1.08, 1.78)	0.0103	0.0323
Yes	0.89 (0.65, 1.23)	0.4869	0.84 (0.39, 1.81)	0.6567	0.84 (0.54, 1.30)	0.4265
Hypertension									
No	1.28 (1.02, 1.61)	0.0327	0.0297	1.43 (0.79, 2.57)	0.2381	0.3300	1.34 (1.07, 1.67)	0.0099	0.1718
Yes	0.92 (0.69, 1.23)	0.5749	0.89 (0.52, 1.52)	0.6798	0.92 (0.69, 1.22)	0.5717

**TG, per SD increase**									
Cardiovascular events									
No	1.24 (1.03, 1.48)	0.0201	0.1182	1.08 (0.76, 1.54)	0.6575	0.9552	1.24 (0.99, 1.54)	0.0593	0.0841
Yes	0.97 (0.70, 1.35)	0.8632	2.13 (0.90, 5.07)	0.0855	0.92 (0.61, 1.40)	0.7000
Hypertension									
No	1.36 (1.12, 1.66)	0.0021	0.0617	1.26 (0.86, 1.83)	0.2344	0.7447	1.36 (1.12, 1.65)	0.0019	0.0529
Yes	0.96 (0.73, 1.26)	0.7772	1.16 (0.71, 1.90)	0.5548	0.96 (0.73, 1.26)	0.7745

**HDL-C, per SD increase**									
Cardiovascular events									
No	1.18 (0.99, 1.41)	0.0597	0.6071	1.02 (0.71, 1.46)	0.9116	0.4121	1.34 (1.06, 1.70)	0.0130	0.6982
Yes	1.36 (0.95, 1.97)	0.0964	0.79 (0.26, 2.37)	0.6717	1.55 (0.98, 2.44)	0.0595
Hypertension									
No	1.34 (1.08, 1.67)	0.0088	0.0293	0.89 (0.51, 1.57)	0.6988	0.3019	1.40 (1.13, 1.73)	0.0021	0.0114
Yes	1.00 (0.76, 1.32)	0.9744	1.23 (0.83, 1.82)	0.3091	1.00 (0.76, 1.32)	0.9842

**LDL-C, per SD increase**									
Cardiovascular events									
No	1.03 (0.85, 1.25)	0.7830	0.6977	0.69 (0.42, 1.14)	0.1499	0.1399	1.09 (0.87, 1.37)	0.4443	0.8265
Yes	1.17 (0.85, 1.61)	0.3249	1.11 (0.56, 2.20)	0.7744	1.12 (0.72, 1.76)	0.6111
Hypertension									
No	1.07 (0.86, 1.33)	0.5176	0.5828	0.89 (0.48, 1.64)	0.7051	0.6185	1.12 (0.91, 1.39)	0.2905	0.7157
Yes	1.04 (0.81, 1.35)	0.7364	0.98 (0.59, 1.61)	0.9345	1.05 (0.81, 1.35)	0.7340

*P-value for test of interaction.

*Data are OR (95% CI). Adjusted for sex (only for total participants), age, smoking status (never/ever or current), alcohol status (never/ever or current), BMI, waistline, physical activity (<30 min a day/0.5–1 h a day/>1 h a day), hypertension, cardiovascular events, metabolic syndrome, family history of hip fracture, blood glucose, blood Ca, calcium and vitamin D supplementation, and T-score for total hip*.

## Discussion

In the present cross-sectional study, there were no associations between per SD increase in TC and LDL level, and an increased risk of osteoporotic fracture in total participants as well as in men and women as individual groups. There was a significant association between per SD increase in HDL-C level and an increased risk of osteoporotic fracture in total participants and in women, whereas no association was observed in men. Additionally, we found a significant association between per SD increase in TG level and an increased risk of osteoporotic fracture in total participants and in women; a nonlinear relationship was observed between per SD increase in TG level and an increased risk of osteoporotic fracture. The risk of osteoporotic fracture in women increased with TG >1.64 mmol/L.

Only few observational studies have investigated the association between serum cholesterol level and fractures (28, 29). A prospective study of 1,396 Swedish participants by Trimpou et al. found that the gradient of risk of serum TC level to predict osteoporotic fracture significantly increases over time (28). Another prospective study of 2062 pre or early perimenopausal women showed that an increase of 50 mg/dL in baseline fasting plasma TG level was associated with a 1.1-fold increased risk of fracture (adjusted HR = 1.11, 95% CI 1.04–1.18). Women with a baseline TG ≥300 mg/dL had an adjusted 2.5-fold greater risk for fractures (95% CI 1.13–5.44) than for women with a baseline TG <150 mg/dL. Moreover, no associations between TC, LDL-C, or HDL-C levels and fractures were observed.

Yamauchi et al. performed a cross-sectional study based on 211 Japanese women (age range, 46–80 years) and suggested that a high-serum LDL-C level may be a risk factor for prevalent non-vertebral fragility fractures, after adjustment for potential confounders (29). In a Turkish observational study with 107 postmenopausal women aged 45–79, per 1-mg/dL increase in TC was found to correlate in a 2.2% decrease in risk of experiencing a vertebrae fracture (22). In a cross-sectional study from a community-based prospective cohort including 289 men, the authors did not observe any association between serum lipids and prevalent vertebral fractures (35). To the best of our knowledge, our study is the first observational studies for older Chinese men and women (aged 55 years or older) to examine the association between serum cholesterol level and osteoporotic fracture. In doing so, we identified several novel and salient features of this relationship.

Many studies have investigated the association between serum TG or HDL-C level and BMD. However, the conclusions between studies are controversial (8, 9, 17–27, 36–40). In a Dutch cross-sectional, population-based study (including 620 men, 635 women aged 65–88 years), men and women in the highest quartile of HDL-C level had a significantly lower BMD compared with men and women in the lowest quartile (37). A large Swedish study of 6,886 women performed by Lidfeldt et al. found a positive correlation between TG level and BMD and a negative correlation between TC or HDL level and BMD (38). Another cohort of 1,289 African ancestry men with a lower serum TG or LDL and higher HDL level exhibited an association with lower trabecular BMD (27). Furthermore, a retrospective analysis of data from 4,613 premenopausal women and 2,661 postmenopausal women aged 20–91 years showed no significant relationship between lipid profiles (TC, TG) and total hip, femoral neck, trochanter, or lumbar spine BMD. Notably, HDL-C level was marginally positively associated with BMD in the lumbar spine (*P* = 0.048) (40). However, another Chinese cross-sectional study of 790 Chinese postmenopausal women found that high HDL-C levels were associated with osteoporosis (OR 1.64, 95% CI 1.16, 2.33, *P* < 0.01), after adjusting for age, menopausal duration, BMI, serum creatinine levels, outdoor activity, smoking, and alcohol intake. Moreover, No association was found between TC, TG, and LDL-C with BMD (39).

Moreover, several meta-analyses reported the relationship between hypolipidemic drugs and osteoporosis (41–44). A recently meta-analysis including 33 studies (16 cohort studies, 7 case-control studies, and 10 randomized controlled trials) showed that statins decreased the risk of total fractures (OR = 0.81, 95% CI 0.73–0.89) and hip fractures (OR = 0.75, 95% CI 0.60–0.92). The use of statins was associated with increased BMD at the total hip [standardized mean difference (SMD) = 0.18, 95% CI 0.00–0.36] and lumbar spine (SMD = 0.20, 95% CI 0.07–0.32) and improved the bone formation marker, osteocalcin (OC) (SMD = 0.21, 95% CI 0.00–0.42) (41).

To date, the pathophysiological links between TG or HDL-C level and osteoporosis as well as related fractures are not entirely clear. The association between serum TG or HDL-C level and osteoporosis may be affected by factors, such as race, ethnicity, age, sex, and menopausal status (8, 45). Previous reports have indicated that oxysterols may stimulate osteogenic differentiation of bone marrow mesenchymal stem cells. Interestingly, HDL-C can remove oxysterols from peripheral tissues and impact the oxysterols acting on the osteogenic differentiation (39, 46).

The study described herein is a cross-sectional study that included 1,791 participants (62.1% postmenopausal women and 213 fractures) aged 55 years and older. This relatively large sample size strengthens the rigor of our findings. We also excluded subjects with kidney disease, cancer/malignancy, diabetes mellitus, rheumatoid arthritis, and thyroid diseases as well as those who had undergone regular treatment for osteoporosis, which may affect lipid and bone metabolism. Furthermore, we considered several confounding variables. Our study also has several limitations. First, as an inherent characteristic of cross-sectional studies, this study cannot draw a causal inference. Further well-designed prospective cohort studies are needed to investigate the relationship identified in this study. Second, recall bias is innate and there is a possibility of confusion of medication history. Third, this study included only Chinese participants aged 55 years or older. Thus, our findings might be applicable to only older Chinese or Asian patients and not to other ethnic groups or a younger population.

Finally, risk of osteoporotic fractures is associated with many factors, such as lifestyle, disease status, metabolic factors, inflammatory states, and genetic factors (4–6, 47, 48). Several fracture risk prediction tools have been developed using different risk factors. These tools include age, height, weight, BMD, osteoporosis treatment, smoking status, alcohol status, and rheumatoid arthritis as the most common risk factors (48). Moreover, vitamin D status and PTH were related to both serum lipids and fractures (49–52). Although wide epidemiologic and clinical covariables were included in the adjustment, we could not exclude the possibility of residual confounding variables in the analyses, such as serum vitamin D status and PTH. Therefore, additional well-designed and stratified cohort studies that include sufficient controls and account for confounding factors are, therefore, needed to elucidate the link between serum cholesterol level and the risk of osteoporotic fracture.

In conclusion, our study demonstrated that, among Chinese older adults, serum HDL-C level is significantly associated with a risk of osteoporotic fractures in women, and serum TG level is significantly associated with a risk of osteoporotic fractures overall and in women with TG level >1.64 mmol/L.

## Ethics Statement

This study was approved by the Ethics Committee of Shanghai Sixth People’s Hospital, and all participants provided written informed consent.

## Author Contributions

YW, SL, and YC contributed to the study design. YW, JD, WZ, CH, and SL performed data collection, data interpretation, and data analysis. SL and YC performed critical review. YW, JD, WZ, CH, SL, and YC performed data collection, case diagnoses, and confirmation of case diagnoses.

## Conflict of Interest Statement

The authors declare that the research was conducted in the absence of any commercial or financial relationships that could be construed as a potential conflict of interest.

## Footnotes

^1^www.empowerstats.com.

^2^http://www.r-project.org.

## Supplementary Materials

Figure S1 Study participants selection flowchart.

Figure S2 Multivariate adjusted smoothing spline plots of serum cholesterol level and fracture in total participants **(A)**, men alone **(B)**, and women alone **(C)**. This model adjusted for sex (only for total participants), age, smoking status (never/ever or current), alcohol status (never/ever or current), BMI, waistline, physical activity (<30 min a day/0.5–1 h a day/>1 h a day), hypertension, cardiovascular events, metabolic syndrome, family history of hip fracture, blood glucose, blood Ca calcium supplement, vitamin D supplement, and T-score for total hip. The red line represents the best-fit line. The blue lines are 95% confidence intervals.
