# Supplementary figures and images for: Association between Serum Cholesterol Level and Osteoporotic Fractures
Source: Front Endocrinol (Lausanne). 2018 Feb 12;9:30. doi: 10.3389/fendo.2018.00030 (PMC5816040; doi:10.3389/fendo.2018.00030)

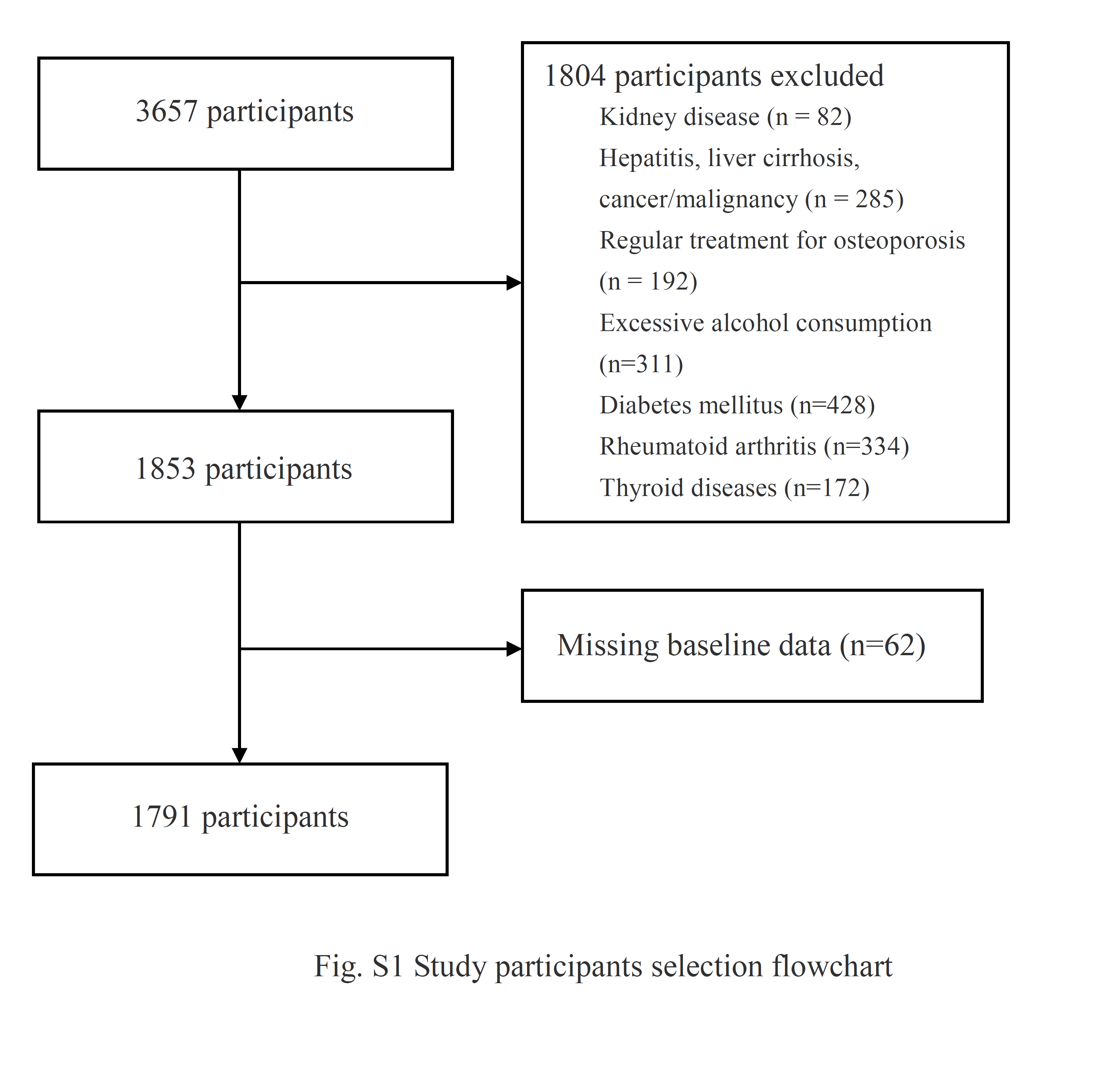

Supplement: Figure S1 — Study participants selection flowchart. [file image_1.tif]

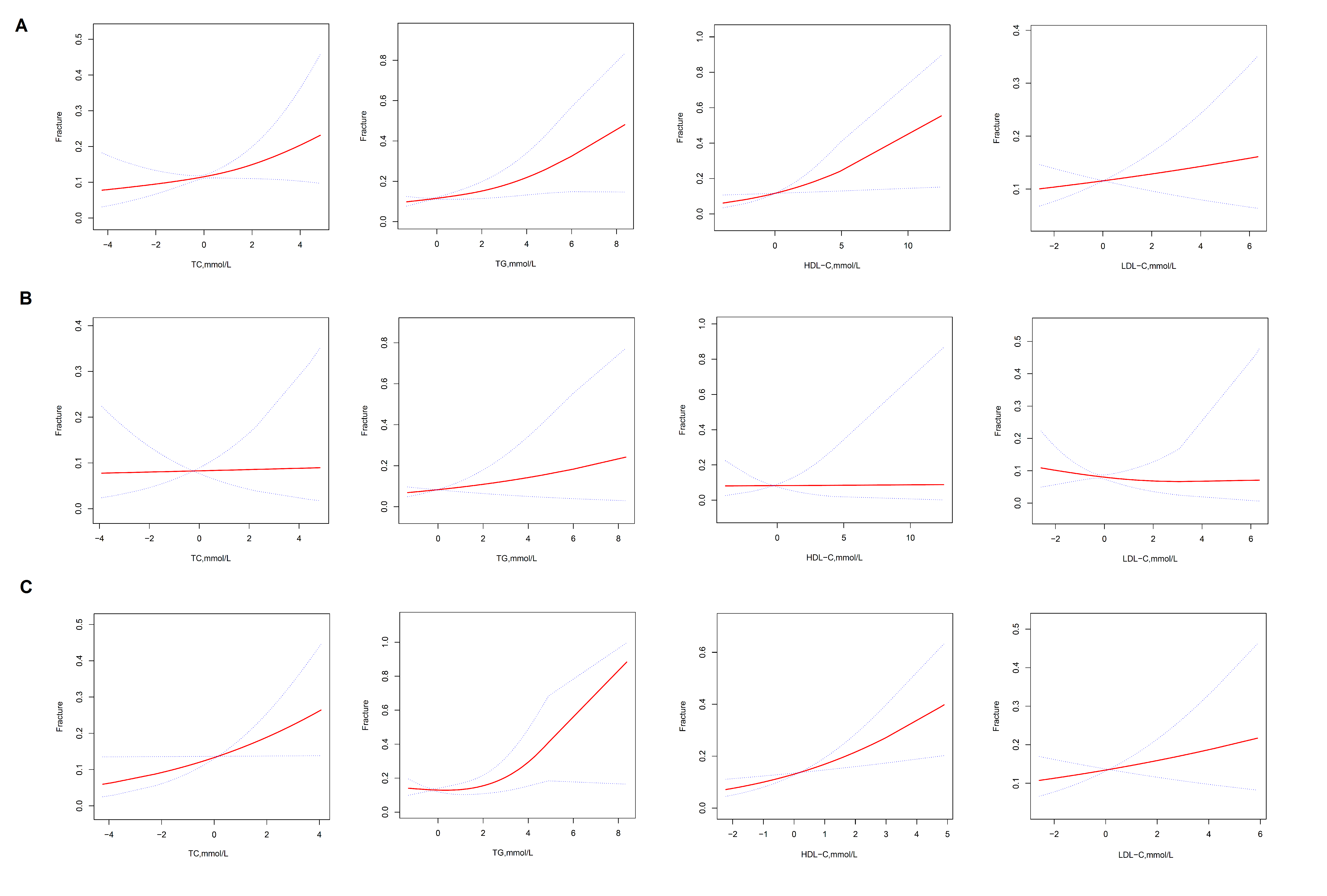

Supplement: Figure S2 — Multivariate adjusted smoothing spline plots of serum cholesterol level and fracture in total participants (A), men alone (B), and women alone (C). This model adjusted for sex (only for total participants), age, smoking status (never/ever or current), alcohol status (never/ever or current), BMI, waistline, physical activity (<30 min a day/0.5–1 h a day/>1 h a day), hypertension, cardiovascular events, metabolic syndrome, family history of hip fracture, blood glucose, blood Ca calcium supplement, vitamin D supplement, and T-score for total hip. The red line represents the best-fit line. The blue lines are 95% confidence intervals. [file image_2.tif]
