# Supplementary material for: Association between Serum Cholesterol Level and Osteoporotic Fractures
Source: Front Endocrinol (Lausanne). 2018 Feb 12;9:30. doi: 10.3389/fendo.2018.00030 (PMC5816040; doi:10.3389/fendo.2018.00030)
Supplement: Supplementary file 3 [file table_1.doc]

|  | **Total participants** | | **Men** | | **Women** | |
| --- | --- | --- | --- | --- | --- | --- |
|  | OR(95%CI) | *P* value | OR(95%CI) | *P* value | OR(95%CI) | *P* value |
| TC (mmol/L) |  |  |  |  |  |  |
| <4.58 | 1.0 |  | 1.0 |  | 1.0 |  |
| ≥4.58, <5.22 | 1.18 (0.72, 1.95) | 0.5072 | 0.86 (0.36, 2.05) | 0.7368 | 1.65 (0.85, 3.22) | 0.1393 |
| ≤5.22, <5.81 | 1.40 (0.86, 2.27) | 0.1799 | 1.02 (0.41, 2.53) | 0.9615 | 1.91 (1.00, 3.65) | 0.0492 |
| ≥5.81 | 1.22 (0.74, 2.01) | 0.4455 | 0.75 (0.27, 2.12) | 0.5907 | 1.68 (0.88, 3.21) | 0.1183 |
| TG (mmol/L) |  |  |  |  |  |  |
| <1.11 | 1.0 |  | 1.0 |  | 1.0 |  |
| ≥1.11, <1.49 | 1.02 (0.63, 1.67) | 0.9255 | 1.01 (0.37, 2.80) | 0.9832 | 1.03 (0.58, 1.82) | 0.9158 |
| ≤1.49, <2.03 | 1.11 (0.68, 1.82) | 0.6748 | 1.21 (0.44, 3.36) | 0.7079 | 1.05 (0.59, 1.87) | 0.8623 |
| ≥2.03 | 1.50 (0.93, 2.42) | 0.0937 | 2.18 (0.86, 5.49) | 0.0997 | 1.22 (0.69, 2.15) | 0.4892 |
| HDL-C (mmol/L) |  |  |  |  |  |  |
| <1.23 | 1.0 |  | 1.0 |  | 1.0 |  |
| ≥1.23, <1.42 | 1.47 (0.86, 2.52) | 0.1625 | 1.64 (0.72, 3.73) | 0.2391 | 1.54 (0.73, 3.26) | 0.2590 |
| ≤1.42, <1.67 | 1.54 (0.91, 2.61) | 0.1080 | 0.78 (0.29, 2.11) | 0.6243 | 1.98 (0.99, 3.98) | 0.0540 |
| ≥1.67 | 1.55 (0.91, 2.65) | 0.1083 | 1.09 (0.36, 3.36) | 0.8762 | 1.99 (1.00, 3.97) | 0.0495 |
| LDL-C (mmol/L) |  |  |  |  |  |  |
| <2.31 | 1.0 |  | 1.0 |  | 1.0 |  |
| ≥2.31, <2.80 | 1.06 (0.65, 1.73) | 0.8076 | 0.87 (0.37, 2.05) | 0.7505 | 1.25 (0.67, 2.33) | 0.4753 |
| ≤2.80, <3.20 | 1.19 (0.73, 1.94) | 0.4789 | 0.78 (0.31, 1.99) | 0.6065 | 1.50 (0.82, 2.76) | 0.1882 |
| ≥3.20 | 0.92 (0.56, 1.49) | 0.7220 | 0.67 (0.25, 1.80) | 0.4254 | 1.02 (0.56, 1.86) | 0.9520 |

Table S1 The association between TC, TG, HDL-C, LDL-C and osteoporotic fracture

Data are OR (95% CI). Adjusted for sex (only for total participants), age, smoking status (Never/ Ever or current), alcohol status (Never/ Ever or current), BMI, waistline, physical activity (<30 min a day/0.5-1 h a day/>1h a day), hypertension, cardiovascular events, metabolic syndrome, family history of hip fracture, blood glucose, blood Ca, calcium and vitamin D supplementation and T-score for total hip.
